# Interleukin-17F expression is elevated in hepatitis C patients with fibrosis and hepatocellular carcinoma

**DOI:** 10.1186/s13027-017-0152-7

**Published:** 2017-07-26

**Authors:** Ming-Sian Wu, Chun-Hsiang Wang, Fan-Chen Tseng, Hsuan-Ju Yang, Yin-Chiu Lo, Yi-Ping Kuo, De-Jiun Tsai, Wan-Ting Tsai, Guann-Yi Yu

**Affiliations:** 1 National Institute of Infectious Diseases and Vaccinology, National Health Research Institutes, 35 Keyan Road, Zhunan, Miaoli County, 35053 Taiwan; 2 Division of Gastroenterology, Tainan Municipal Hospital, Tainan, 70173 Taiwan; 3 Center of Infectious Disease and Signaling Research, National Cheng-Kung University, Tainan, 70101 Taiwan

**Keywords:** Il-17F, Hepatitis C virus, Fibrosis, Hepatocellular carcinoma

## Abstract

**Background:**

The role of interleukin (IL) 17A in chronic liver diseases had been extensively studied, but the function of IL-17F, which shares a high degree of homology with IL-17A, in the progression of chronic hepatic diseases is poorly understood. The aim of the study was to evaluate the association between IL-17F and liver diseases including, fibrosis and hepatocellular carcinoma (HCC).

**Methods:**

Hepatic tumor samples from both hepatitis C virus (HCV) positive and negative patients (without HBV and HCV, NBNC) were examined with quantitative PCR and immunohistochemistry staining for inflammatory cytokine genes expression. In addition, 250 HCV patients naïve for interferon treatment were also subjected to enzyme-linked immunosorbent Assay (ELISA) for their serum cytokine concentrations.

**Results:**

Serum IL-17F concentrations were significantly elevated in HCV patients with severe fibrosis stages. In accordance with serum data, IL-17F expression was also found higher in HCV-associated HCC tissues compared with NBNC HCC tissues at both the mRNA and protein levels.

**Conclusions:**

Our data suggest that IL-17F might be used as a valuable biological marker than IL-17A during chronic fibrosis progression and HCC development in HCV patients.

## Background

Approximately 80 million people worldwide have viremic hepatitis C virus (HCV) infection, which causes chronic hepatitis, fibrosis, cirrhosis, and hepatocellular carcinoma (HCC) [1]. The full progression of end-stage liver diseases in HCV-infected patients takes about two to three decades, which provides a window for intervention. Liver fibrosis is a protective response to chronic hepatic injury that leads to accumulation of extracellular matrix proteins [2]. Extensive fibrosis may result in cirrhosis and, in severe cases, lead to liver failure requiring liver transplantation. Severe fibrosis and cirrhosis are the outcome of continuous liver injuries, which can be caused by chronic virus infection or long-term alcohol consumption. Liver fibrosis is one of the known risk factors for HCC [3]. Chronic inflammation caused by innate and adaptive immune responses to HCV infection is involved in the progression of HCV-associated diseases, such as liver cirrhosis and HCC [4, 5]. Identification of factors involved in HCV pathogenesis or biomarkers associated with liver diseases may provide new intervention and treatment approaches for HCV-related diseases.

T helper (Th) 17 cells are a subset of CD4^+^ T cells that mediate a protective role against bacterial and fungal infections [6, 7] as well as a pathological role in inflammation-associated diseases, such as autoimmune diseases and cancer [8–10]. Transforming growth factor (TGF)-β, interleukin (IL)-6, IL-21, and IL-23 are the key cytokines for Th17 cell maturation and production of IL-17 family cytokines, the secretion of which is a quintessential defining feature for Th17 cells [11–13]. The IL-17 family has six members, including IL-17A–F. IL-17A and IL-17F have high sequence homology and are expressed as homodimers or as an IL-17A + F heterodimer to induce expression proinflammatory cytokines, chemokines, antimicrobial peptides, and matrix metalloproteinases in IL-17 receptor-bearing cells [14].

Th17 cells play an important role in many liver diseases, such as liver fibrosis, alcoholic liver disease, chronic hepatitis B, and autoimmune liver disease [15–18]. The Th17 cell population is increased in chronic hepatitis C patients and Th17 cell abundance correlates positively with liver injury but inversely with HCV RNA load [19]. Although IL-17A had been extensively studied in chronic liver diseases, the function of IL-17F, which shares a high degree of homology with IL-17A, in the progression of chronic hepatic diseases is poorly understood. IL-17F can be secreted by CD8^+^ T cells, γδ T cells, NKT cells, LTi-like cells, and epithelial cells in addition to being secreted by Th17 cells (CD4^+^ T cells) [8, 20]. IL-17F has a weaker receptor binding affinity than IL-17A and therefore induces less expression of proinflammatory cytokines than IL-17A [21]. The aim of this study was to evaluate the association between the expression of IL-17F and HCV-associated diseases by the evaluation of Th17-related cytokine expression in HCC tissue and serum from HCV patients.

## Methods

### HCC cohort

RNA samples derived from cancerous and non-cancerous parts of HCC tissue from patients infected with HCV and controls negative for both HBV and HCV (“non-B, non-C”, NBNC) were obtained from the Taiwan Liver Cancer Network (TLCN) [22].

### Treatment naïve HCV cohort

A total of 250 patients who had serologically demonstrated HCV infection and were treated at Tainan Municipal Hospital (TMH), Tainan, Taiwan from 2003 to 2008 were included in our serum cytokine analysis. Baseline sera were collected from the patients within two months before they underwent HCV antiviral therapy. Patients infected with HBV or HIV were excluded from the study. The diagnoses of hepatic steatosis, fibrosis, and HCC of the HCV cohort were independently confirmed by two pathologists at TMH. Based on the METAVIR grading system, liver fibrosis status was classified as: no fibrosis (F0), portal fibrosis without septa (F1), with few septa (F2), with numerous septa but without cirrhosis (F3), and with cirrhosis (F4).

### Serum cytokine detection

IL-6, IL-17A, IL-17F, and IL-21 concentrations in serum samples were measured by enzyme-linked immunosorbent assay (ELISA) according to the manufacturer’s instructions. Human IL-6, IL-17A, and IL-17F ELISA kits were purchased from R&D Systems (MN, USA), and the IL-21 immunoassay kit was obtained from eBioscience (CA, USA). The kits’ analytic sensitivities were 9.375 pg/ mL for IL-6, 15.625 pg/mL for IL-17A, 312.5 pg/mL for IL-17F, and 8 pg/mL for IL-21.

### Cytokine mRNA quantification

Total RNA samples (1 μg) extracted from HCC cancerous and non-cancerous tissues were used for cDNA synthesis with SuperScript-III First-Strand Synthesis System (Invitrogen, Carlsbad, CA). cDNAs were then used as templates in the quantitative-polymerase chain reaction (PCR) with gene specific primers and SYBR green dye to determine quantification cycle (Cq) by Applied Biosystems 7900HT Fast Real-Time PCR System. Primer sequences for human cytokine IL-17A and IL-17F and housekeeping gene ubiquitin C (UBC) [23] were listed in Table 1. Relative cytokine mRNA expression level was normalized to UBC reference gene by the 2^-ΔΔCq^ method [24]. Cytokine mRNA expression with Cq < 35 cycles was counted as positive, and the positive frequency was determined for cancerous and non-cancerous tissue separately for each group.

**Table 1:** Primer sequences for quantitative RT-PCR

Gene Name	Oligo sequences
UBC forward	5′ CCTGGTGCTCCGTCTTAGAG 3’
UBC reverse	5′ TTTCCCAGCAAAGATCAACC 3’
IL-17A forward	5′ AATCTCCACCGCAATGAGGA 3’
IL-17A reverse	5′ ACGTTCCCATCAGCGTTGA 3’
IL-17F forward	5′ GAAGCTTGACATTGGCATCA 3’
IL-17F reverse	5′ GATGCAGCCCAAGTTCCTAC 3’

### IL-17F immunohistochemistry

Paraffin-embedded sections of the HCC cohort were subjected to immunohistochemistry (IHC) by following the protocol described previously with minor modification [25]. In brief, paraffin-embedded sections were deparaffinized and incubated in citrate buffer (pH 6) with 0.05% Tween 20 at 95–98 °C for 20 min. After 3% H_2_O_2_ and 1% bovine serum albumin blocking, slides were incubated with the anti-IL-17F antibody (ab168194, Abcam) overnight at 4 °C, followed by incubation with a secondary antibody (EnVision^+^ system-HRP-labeled polymer, DakoCytomation) at room temperature for 1 h. The sections were then stained with 3,3′-diaminobenzidine substrate and counterstained with hematoxylin. Samples were considered IL-17F-immunopositive if at least 10% of randomly selected fields contained positive staining signals.

### Statistical analyses

Statistical analyses were performed with SPSS software (IBM, NY, USA). Serum cytokine and mRNA expression were across-analyzed with demographic and clinical groups using the Mann-Whitney U test and Kruskal-Wallis tests. The relative distribution of IL-17A and IL-17F mRNA expression in cancerous and non-cancerous tissue was analyzed by the Fisher exact test and Mann-Whitney U test. In all cases, *p* < 0.05 was considered statistically significant.

## Results

### IL-17F expression was elevated in HCV-associated HCC

Table 2 listed that the characteristics of the HCV (*N* = 40) and NBNC (*N* = 32) cancer patients from whom paired cancerous and adjacent non-cancerous tissues from TLCN tissue bank were obtained and subjected to quantitative RT-PCR analysis with SYBR dye for IL-17A and IL-17F mRNA detection. IL-17A mRNA was present in more than 75% of RNA samples derived from both cancerous and non-cancerous tissues. In contrast, IL-17F mRNA was expressed only in a portion of HCC samples. As shown in Table 2, the positive frequency was higher in cancerous tissue than in non-cancerous tissue, and notably higher in HCV-infected tissue (47.5% vs. 15%, *p* = 0.003 by Fisher exact test) than in NBNC patients (40.6% vs. 18.8%, *p* = 0.1 by Fisher exact test). Relative IL-17A mRNA expression level demonstrated no differences between cancerous and non-tumor counterpart tissues either derived from HCV-infected patients or NBNC patients. The relative IL-17A and F mRNA expression level in those positive cases did not show a significant difference between HCV and NBNC patients.

**Table 2:** Demographic Summary of TLCN HCC cohort

Variable	HCV (*n* = 40)	NBNC (*n* = 32)
Age	66.3 ± 8.4 (59.3–72.0)	66.0 ± 14.9 (61.0–76.0)
Gender	Male:32, Female:8	Male:22, Female:10
Presence of cirrhosis	45.0%	21.9%
Tumor size (cm)	4.5 ± 2.3 (3.0–5.9)	8.0 ± 5.1 (3.5–11.8)
Positive frequency^a^	(Tumor, Non-Tumor)	(Tumor, Non-Tumor)
IL-17A	80.0%, 75.0%	87.5%, 87.5%
IL-17F	47.5%, 15.0%	40.6%, 18.8%

^a^Specific cytokine gene expression was detected by quantitative RT-PCR

**Fig. 1:**
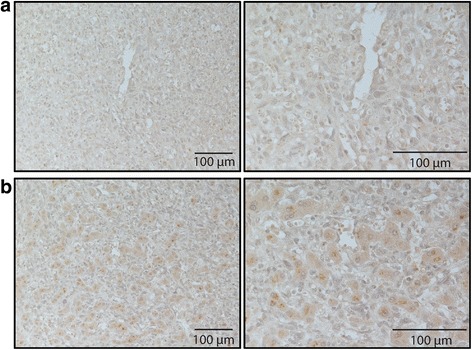
Representative images of IL-17F immunohistochemistry staining. Representative images for IL-17F IHC labeling in HCV-infected HCC tumor tissue samples without (**a**) and with (**b**) IL-17F immunopositivity. Magnification in the left column-200X and in the right column-400X

IHC revealed IL-17F-immunopositive signals in hepatocytes in HCC tissues (Fig. 1). In total, 85% of the HCC tumor samples from the HCV group and 62% of the HCC tumor samples from NBNC group were positive for IL-17F staining (*p* = 0.0002 by Fisher exact test). In summary, IL-17F expression was higher in HCV-associated HCC tissues compared with NBNC HCC tissues at both the mRNA and protein levels.

### Association of serum IL-17F level with liver fibrosis progression in treatment naïve HCV patient cohort

Due to HCC development is largely confined to patients with liver fibrosis and cirrhosis, a retrospective cohort study was further conducted to determine serum IL-17F expression levels in HCV patients with chronic hepatic diseases. Diagnosis of HCV infection was confirmed in all 250 HCV patients by two sequential tests for HCV antibodies, followed by an HCV RNA assay. The data consisted of 127 men (51%) and 123 women (49%) with a mean age of 56.6 years; 39.2% of the patients had steatosis, 100% had fibrosis, and 15.2% had HCC (Table 3). The distribution of liver fibrosis stage in this cohort was as follows: F1, 7 cases (2.8%); F2, 102 cases (40.8%); F3, 94 cases (37.6%); and F4 (cirrhosis), 47 cases (18.8%). As shown in Table 3, IL-17F ELISAs of 250 serum samples obtained from treatment-naïve HCV patients yielded a mean serum IL-17F concentration of 1246.7 pg/ml. Among the 250 samples, 76 (30.3%) had IL-17F level > 312.5 ng/mL, the lower detection limit of the ELISA. The expression of inflammatory cytokines, IL-17A, IL-21, and IL-6, which are functionally associated with IL-17F were also measured by ELISA. As some serum samples collected from the HCV patients in 2003–2008 were not sufficient for addition ELISAs, the case numbers (N) for these cytokines were less than 250. IL-17A was detected in very few samples (9/144, 6.3%) at a low concentration (mean: 4.3 pg/mL). IL-21 was detected in 82/137 samples (59.9%) at a mean concentration of 73.2 pg/mL, and IL-6 was detected in 31/153 samples (20.3%) at a mean concentration of 50.6 pg/mL.

**Table 3:** Variables of HCV infected patients from TMH

Variable		N	Mean (IQR)	Positive rate
Age		250	56.6 ± 10.5 (51.5–64.8)	
Gender		Male:127, Female:123		
Steatosis		98 (39.2%)		
Fibrosis	F0	0 (0%)		
	F1	7 (2.8%)		
	F2	102 (40.8%)		
	F3	94 (37.6%)		
	F4	47 (18.8%)		
HCC		38 (15.2%)		
IL-17F		250	1246.7 pg/mL (0–877.7)	30.4%
IL-17A		144	4.3 pg/mL (0–0)	6.3%
IL-21		137	73.2 pg/mL (0–84.7)	59.9%
IL-6		153	50.6 pg/mL (0–0)	20.3%

**Fig. 2:**
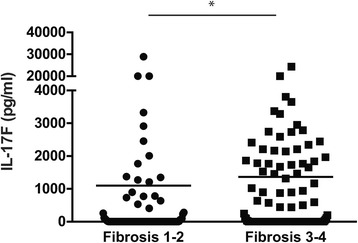
Association of serum IL-17F level and fibrosis stages in HCV patients. Distribution of serum IL-17F level by fibrosis stages (F1–2 vs. F3–4) in HCV patients. * *p* < 0.05, Mann-Whitney U test

The correlation efficiency between serum IL-17F levels and chronic hepatic diseases, including hepatitis, steatosis, fibrosis, and HCC was evaluated as shown in Table 4. Serum alanine aminotransferase (ALT) which is usually elevated during liver inflammation was used as an index for hepatitis in Table 4. When the patients were separated into mild (F1 and F2) and severe (F3 and F4) fibrosis groups for statistical analysis, serum IL-17F level was correlated with fibrotic severity (rho = 0.127, *p* = 0.045) (Fig.2). Levels of cytokines IL-17A and IL-21 had strong (rho = 0.547, *p* < 0.0001) and moderate (rho = 0.218, *p* = 0.011) correlations, respectively, with IL-17F in the HCV patients. However, serum IL-17A and IL-21 level were not significantly associated with fibrosis or other HCV-associated diseases by statistical analyses.

**Table 4:** Correlations of serum IL-17F level with the HCV infection-associated liver diseases and other proinflammatory cytokines

		ALT	Steatosis	Fibrosis^c^	HCC	IL-17A	IL-21	IL-6
IL-17F	Correlation Coefficient (r)	−0.094	0.012	0.127^a^	0.014	0.547^b^	0.218^a^	0.009
	Sig.	0.142	0.850	0.045	0.826	0.000	0.011	0.912

^a^Correlation is significant at the 0.05 level (2-tailed)

^b^Correlation is significant at the 0.01 level (2-tailed)

^c^ Fibrosis: stage >2

Spearman’s rank correlation

## Discussion

The present study showed that a higher percentage of HCV-associated HCC tissues than of NBNC-HCC tissues were found to have IL-17F mRNA expression. IL-17F mRNA expression was higher in tumor tissue than in non-tumor counterparts. Furthermore, the serum IL-17F levels were elevated in HCV-infected patients with severe liver fibrosis than in patients with mild liver fibrosis.

Chronic HBV, HCV infection and steatohepatitis are common causes of liver fibrosis [26–28]. The relevance of Th17 and IL-17 in liver fibrosis has been studied in human patients and mouse models [29, 30]. Chang et al. showed that circulating and liver-infiltrating Th17 cell levels correlate with severity of liver inflammation [19]. However, serum IL-17A levels are low in HCV patients and appear not to correlate with HCV-related fibrosis [31]. In our HCV cohort study, the mean serum IL-17F level was much higher than that of IL-17A. The severity of liver fibrosis in HCV patients was associated only with IL-17F, but not with IL-17A, suggesting that IL-17F might be a better biomarker than IL-17A for HCV-associated fibrosis progression. The serum IL-17F level did not have a significant correlation with serum alanine aminotransferase activity in the HCV cohort, suggesting that IL-17F elevation was not associated with acute liver inflammation.

IL-17A and IL-17F have highly homologous amino acid sequences, bind the same receptor, and activate similar proinflammatory responses, but these two cytokines differ in tissue distribution and receptor binding affinity [32]. In the present HCV cohort, more serum samples were positive for IL-17F than with IL-17A, and the mean IL-17F level was much higher than the mean IL-17A level. High levels of IL-17F can be due to a high transcription activity, a long protein half-life, or production by different cell types. Given that the expression of serum IL-17A and IL-21, two key cytokines produced by Th17 cells, was associated with IL-17F expression, it is likely that Th17 cells contribute to IL-17F expression. However, in our IL-17F IHC analysis of HCC tissue sections, IL-17F expression was found mainly in hepatocytes. Serum IL-17F in HCV-infected patients may be produced partially by hepatocytes. The stimuli capable of triggering IL-17F expression in hepatocytes and other cell types and the responses activated by IL-17F-dependent signaling in the liver will need to be addressed further.

A pathological role of IL-17 and Th17 in HCC development has been proposed based on recent studies [29, 33–35]. Proinflammatory, anti-apoptosis, and pro-angiogenesis signals may contribute to the tumor promotion role of Th17 cells and IL-17 in HCC development [29]. The role of IL-17F in HCC remains undetermined. In the present study, we found that IL-17F was elevated in patients with HCV and advanced fibrosis and that IL-17F mRNA was also elevated in HCV-associated tumor tissue. Notably, the IL-17F protein was found mainly in hepatocytes. The exact function of IL-17F in HCC development needs to be clarified with long-term follow-up cohort study.

## Conclusions

The importance of IL-17F in chronic hepatic diseases was poorly investigated; however, we had found Th-17 associated inflammatory cytokine, IL-17F, which took a major part in severe liver fibrosis and HCC symptoms in HCV patients than other inflammatory cytokines, IL-6, and IL-17A. It appears that IL-17F can be a precise biomarker for the diagnosis of liver fibrosis and HCC progression in the future.
